# The effect of exercise on prescription on physical activity and wellbeing in a multi-ethnic female population: A controlled trial

**DOI:** 10.1186/1471-2458-12-758

**Published:** 2012-09-10

**Authors:** Maaike GJ Gademan, Marije Deutekom, Karen Hosper, Karien Stronks

**Affiliations:** 1 Department of Public Health, Academic Medical Center, Meibergdreef 9, Postbus 22660, 1100 DD Amsterdam, The Netherlands

**Keywords:** Exercise referral program, Deprived neighborhoods, Physical activity

## Abstract

**Background:**

In Western countries, individuals from multi-ethnic disadvantaged populations are less physically active than the Western population as a whole. This lack of physical activity (PA) may be one of the factors explaining disparities in health. Exercise on Prescription” (EoP), is an exercise program to which persons are referred by primary care. It has been developed to suit the needs of physically inactive women from diverse ethnic backgrounds living in deprived neighborhoods in the Netherlands. The effectiveness of this program has however, not yet been proven.

**Methods:**

A total of 514 women from diverse ethnic backgrounds were included in this study (192 EoP, 322 control group). Women in the EoP group participated in 18 sessions of supervised PA. The control group received care as usual. At baseline, 6 and 12 months the women attended an interview and a physical examination. Outcome measures were PA, BMI, weight circumference, fat percentage, oxygen uptake, mental well-being, subjective health and use of care.

**Results:**

Of the participants 59% had a low educational level and 90% of the women were overweight or obese. Compliance was high, only 14% dropped out during the course of the program. Total PA did not change, PA during leisure time increased at 6 and at 12 months and PA during household activities increased at 12 months (P_EoPvsControl_ < 0.05). EoP had no significant effect on the other outcome variables.

**Conclusions:**

EoP was successful in recruiting its target population and compliance was high. The effect of EoP on PA, health and mental well-being was limited. In this format EoP does not seem to be effective for increasing PA and the health status of non-Western migrant women.

**Trial registration:**

Dutch Trial register: NTR1294

## Background

One of the biggest public health problems of the 21^st^ century has become physical inactivity [1]. It is an important risk factor for cardiovascular disease, diabetes and several kinds of cancers. At least 60% of the global population fails to achieve the minimum recommendation of 30 minutes daily moderate intensity physical activity (World Health Organization, 2010). In Western countries, individuals from multi-ethnic disadvantaged populations are even less physically active than the Western population as a whole [2,3]. This lack of physical activity may be one of the explanatory factors for disparities in health. For instance, in the Netherlands, Turkish, Moroccan and Surinamese women are more often overweight and suffer more frequently from diabetes mellitus, hypertension, and mental health problems than Dutch women in the same age group – they also rate their own health poorer [4-7].

Exercise referral schemes (ERS) were developed to promote physical activity levels in people with signs of lifestyle diseases. In ERS, patients are referred by their general practitioner to a supervised exercise program. The aim of these programs is to eventually change a physical inactive lifestyle into an active one. Although long-term effects of ERS are inconsistent, physical activity (PA) can be improved by these interventions [8,9]. In terms of health status, ERS seem to be able to improve self reported health [10-12]. Knowledge on the effects of ERS on aerobic fitness, body mass index (BMI), body fat percentage, cholesterol levels and blood pressure is limited and effects differ over studies [10,13,14].

Effects of ERS may differ by socioeconomic and ethnic characteristics of the population as cultural differences and practical problems such as language barriers make it more difficult to communicate with these groups in health promotion activities. Also, unique barriers to PA have been repeatedly shown in women from different ethnic backgrounds. For example, Muslim women prefer an all-female environment for PA [15]. However, no studies specifically report on the effects of ERS in socio-economically deprived populations or ethnic minority groups. More specifically, it is not known what the effects of these programs are in these targets group on behavioral changes towards PA or how this program influences objective health level (fitness, body size) and subjective health level (perceived health and well-being).

Therefore, the aim of our study was to evaluate the effect of EoP in physical inactive women living in multi-ethnic deprived neighborhoods in the Netherlands. This study was based on the program “Exercise on Prescription” (EoP). This program has been shown to be successful in reaching physical inactive women from diverse ethnic backgrounds who are living in deprived neighborhoods [15,16]. The protocol of the evaluation study has been published before [17].

## Methods

### Study design

This study was approved by the Medical Ethics Committee of the Academic Medical Center of Amsterdam, and was registered in the Dutch Trial Register (NTR1294). It was originally set up as a randomized controlled trial comparing referral to an exercise program (EoP) with usual care. Details about the study design have been published elsewhere [17]. All participants gave informed consent.

### Study population and recruitment

Eligible for the trial were women, aged 18 to 65 years from ethnic minority groups who visited their GP regularly, and were physically inactive according to the subjective assessment of their GP. Exclusion criteria were, participation in EoP in the year preceding the start of inclusion, pregnancy, diagnosis or treatment of a disorder that makes PA impossible or planned emigration or a long-term stay abroad.

As described in the protocol [17], we originally recruited four general practices situated in deprived neighborhoods in the Dutch city of the Hague. From their patient registries, eligible women were pre-randomized into the EoP or control group. These women received a letter from their GP. After several months, it appeared that recruitment by the GP of our randomized participants went very slowly. Although the GPs who participated in our trial were familiar with EoP and also had a positive attitude towards the program, inclusion to the intervention group appeared to be insufficient. The main barriers faced by the GPs were the lack of time and the lack of attention needed to inform patients about the study. We therefore decided to leave our initial protocol and to make use of the natural patient flow of the EoP program for our intervention group. Following the intake at EoP these patients were informed about the study and were asked for their participation. This implies that during the process of recruiting participants, the design of our study was changed to a non-randomized controlled trial. Further details of the process of recruiting participants can be found in the study protocol [17].

**Figure 1:**
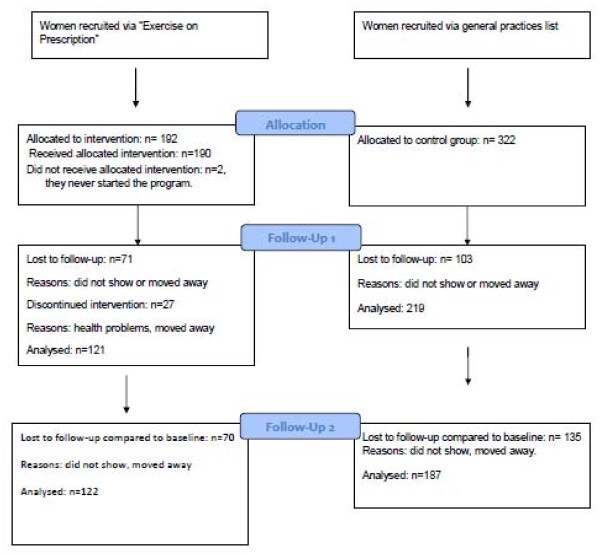
Flowchart.

In total 514 women were included, 192 in the EoP group and 322 in the control group (Figure 1). The number of women in the control group was larger than in the intervention group, because we anticipated the dropout rate in this group to be much higher than in the intervention group. Eventually, the dropout rate in the control group appeared to be lower than we anticipated, probably due to specific measures that we have taken (visit at home, several attempts to contact people etc.).

### Intervention

EoP was set up to promote physical activity among inhabitants of deprived neighborhoods in the Hague. The purpose was to let them experience the beneficial effects of sports and to stimulate them in such a way that they would be able to continue this behavior after completion of the program. EoP consisted of an intake followed by 18 sessions of supervised physical activity and a final evaluation. Training sessions were held once a week. EoP offered Fitness, Aquarobics, Aerobics and Dancing. The personal coaching during EoP was organized in two parts. The first nine supervised sessions aimed at motivating the participant through increasing awareness of the positive effects of exercise. The following nine sessions were dedicated to empowering the participant with respect to the continuation of the healthy (physical) behavior. During the sessions, the participants were given individual advice as to how to reach the goal of exercising 30 minutes a day for at least 5 days a week, either by increasing daily physical activity (walking etc.) or by further participating in sports - whether organized or not. In addition, experienced social support, attitudes towards sports and ways of coping with (negative) feedback from the community was addressed. This supervision and coaching was done by specially trained sports instructors, under the responsibility of the lifestyle advisor. More details on the design of the intervention and the rationale behind this have been given elsewhere [15-17].

#### Final evaluation with lifestyle advisor and further referral

After the first 18 weeks, a final evaluation took place with the lifestyle advisor in which the individual’s achievements, further goals and experiences in the program were evaluated. At the same time, the lifestyle advisor could refer the participants to “Exercise without Prescription”. Exercise without Prescription was designed to address a further aspect that was deemed important by participants in the process evaluation of EoP, namely that participants experienced difficulty in continuing the desired behavior after the EoP program.

### Control group

Women in the control group received care as usual and were only approached by a professional interviewer for an interview on lifestyle (in their preferred language wherever possible) and measurements. Per interview, participating women both in the control and intervention group received a voucher of 10 Euros to stimulate participation in the study.

### Data collection

Data collection took place at baseline (directly following the EoP intake), after 6 months (shortly after completion of EoP) and after 12 months (follow-up measurement 6 months after completion of EoP) for both the intervention group and the control group. During this measurement an interview and wherever possible a physical examination took place.

#### Interview

Data on self reported physical activity and secondary outcome measures were collected during a structured interview, which was conducted by a trained female bilingual interviewer with a Surinamese, Moroccan or Turkish background. As far as possible, participants saw the same interviewer for each session. During the interview a questionnaire was used which had been constructed to measure self reported physical activity (our primary outcome measure), subjective and objective health, and health care use. Where possible, standard validated questionnaires that have been used previously among ethnic minority populations were used [7,18]:

##### Self-reported physical activity

· Self reported physical activity related to commuting, household, work, leisure time, and sports was measured with the short questionnaire to asses health enhancing physical activity (SQUASH), which has been used previously among Turkish, Moroccan and Surinamese populations [3,7,18,19]. Dancing was added to the leisure time domain of the questionnaire since it is a common activity among migrant women. The SQUASH covers similar topics to the long-format International Physical Activity Questionnaire (IPAQ) [20].

For each domain, participants were asked to specify

the frequency (times per week),

intensity (light, moderate or vigorous) and

duration per day.

Given the difficulty of answering questions on level of intensity, the questions concerning work and household related physical activities in the SQUASH are predefined into two questions: one question about the frequency, intensity and duration for *light* work/household physical activities and one question about *vigorous* work/household physical activities [19]. The total minutes of activity were calculated by multiplying frequency (days/week) by duration (min/day) of physical activity. Activity scores for separate questions were calculated by multiplying total minutes of activity by the intensity score. The intensity score was expressed in METs (i.e. metabolic equivalent or number of times resting metabolic rate). One MET equals the resting metabolic rate obtained during quiet sitting and equals an approximate oxygen uptake of 3.5 ml/kg/min. The oxygen expenditure for physical activities ranges, for example, from 0.9 MET for sleeping to 16 METS running a 6 minute mile. All activities were coded according to the Ainsworth Compendium of Physical Activities [21].

##### Subjective health measures

· Perceived health was measured with one item on self-perceived general health with a 5-point likert scale ranging from very good to very poor.

· Well-being was measured with the W-BQ12 (well-being questionnaire)[22].

##### Self-reported use of care

· Use of care was assessed according to questions about the frequency of health care use in the last two months.

#### Physical examination (objective health measures)

The physical examination consisted of a standardized measurement of weight, height, fat percentage, waist circumference and physical fitness level. Total body fat percentage (bioelectrical impedance method), and weight (to the nearest 200 grams) were measured on an electronic scale (TANITA UM-070 and SECA 877 respectively). Height (to the nearest 0.01 meter) and waist circumference (midway between the lower rib margin and the iliac crest) were measured with a tape measure. Physical fitness (estimated maximal oxygen uptake) was assessed with the Siconolfi step test [17,23]. During this test, participants were asked to step up and down a portable bench for three minutes at a rate of 17 steps per minute. This pace was held constant with the help of a metronome. Heart rate was monitored continuously by an Iventum SH100 heart rate monitor (Bilthoven, The Netherlands) and was recorded at the end of the stage.

### Data analysis

Unpaired Student t-tests were used to assess baseline differences between groups for continuous variables and Chi-Square was used for categorical variables. To account for dependencies of observations within the participants we applied linear mixed models: assuming a Gaussian distribution in case of continuous responses, a logistic distribution in case of binary/ordinal responses. We applied a random intercept so that all data could be included irrespective of whether data for each person at all three time points was available. The total activity score and the activity scores for separate questions were used in the statistical analysis. Statistical analysis was performed in R 2.13.1. *P* values < 0.05 were considered statistically significant.

## Results

### Characteristics of participants

Baseline characteristics are depicted in Table 1. Overall the educational level was low, 59% of participants had a low educational level or no education at all. Furthermore, 90% of the women were overweight or obese (Table 1). As such, EoP was successful in including its target population. After analyzing the characteristics of the two groups, we found significant differences between them at baseline. Women in the EoP group were older, heavier, had a higher fat percentage and waist circumference, a poorer mental well-being and a poorer subjective health; also ethnic background was distributed differently (Table 1). Therefore, in further analysis, corrections were made for age, BMI, ethnicity and mental well-being.

**Table 1:** Baseline characteristics

	**EoP-group**	**N**	**Control Group**	**N**	***P***
Age (years)	45 ± 10	189	41 ± 12	311	0.01
Ethnicity		189		311	0.00
Turkish	49 (26%)		152 (49%)		
Surinamese	54 (29%)		34 (11%)		
Moroccan	38 (20%)		48 (15%)		
Other	48 (25%)		77 (25%)		
Education		154		294	0.45
Primary school	45 (29%)		89 (30%)		
Secondary school	34 (22%)		69 (24%)		
Higher education	27 (18%)		55 (19%)		
No education	48 (31%)		81 (28%)		
BMI (kg/m^2^)	32 ± 6	183	29 ± 5	172	0.01
Weight					0.00
Healthy weight (BMI ≤ 25)	20 (11%)		42 (24)		
Overweight (BMI = 25–30)	54 (29%)		56 (33)		
Obese (BMI ≥ 30)	109 (60%)		74 (43)		
Fat percentage (%)	41 ± 6	177	37 ± 7	167	0.01
Waist circumference (cm)	102 ± 13	156	94 ± 14	161	0.03
Maximal oxygen uptake (ml/kg/min)	23.5 ± 6.5	100	25.4 ± 6.4	129	0.02
Subjective health		189		311	0.00
(very) Good	21 (11%)		90 (28)		
Fair	83 (44%)		140 (44)		
(very) Poor	85 (45%)		81 (25)		
Mental well-being	17.9 ± 8	174	20.4 ± 8	297	0.01

Table 1. BMI: body mass index; EoP: Exercise on Prescription. The Hague, 2008–2010.

### Drop out

A total of 192 women were referred to EoP, of these, 2 women never started the program. In both cases the reason for not starting EoP was admission to hospital for an operation (Figure 1). Of the 190 women who did start the program, 27 women (14%) dropped out during its course. Reasons for dropping out were diverse, varying from health problems to moving to a different place. The women who finished the program (86%) attended almost all 18 sessions.

### Lost to follow-up

Of the 190 women in the EoP group, 121 (64%) women attended the second and, 122 (64%) the third measurement (d 1). Hence 36% were lost to follow-up for both measurements. In the control group, 219 women (68%) were present at the second and, 187 (58%) at the third measurement (Figure 1).

### Physical activity

We found a difference between the groups at baseline in the total volume of conducted PA, the control group was physically more active than the EoP group. Total PA scores are given in Table 2. Both at short-term and at long-term, EoP had no effect on the total amount of PA per week. When investigating the different exercise domains (work, household, leisure time), the following effects were found.

**Table 2:** Changes in the Exercise on Prescription and the Control group

	**EoP group intercept**	**Δ 6 months**	**Δ 12 months**	**Control group**	**Δ 6 months**	**Δ 12 months**	**Δ change 6 months between groups**	**Δ change 12 months between groups**
Physical activity								
Total score	3147 (2473, 3822)	285 (−305, 875)	−88 (−660, 485)	4508 (3898, 5118)	108 (−466, 681)	−430 (−1025, 166)	177 (−646, 1001)	342 (−485, 342)
Leisure time score	854 (647, 1062)	65 (−155, 285)	46 (−168, 261)	1296 (1143, 1449)	−251 (−412, -89)*	−386 (−556, -215)*	316 (43, 589)*	432 (158, 706)*
Household score	2123 (1867, 2380)	118 (−110, 346)	258 (35, 482)*	2441 (2216, 2667)	−22 (−238, 193)	−54 (−279, 170)	140 (−174, 454)	312 (−4, 629)
Work score	750 (237, 1261)	−99 (−590, 390)	−490 (−966, -13)*	815 (438, 1191)	250 (−108, 609)	−191 (−571, 190)	−350 (−957, 258)	−299 (−909, 311)
Commuting score	93 (54, 132)	60 (8, 111)*	7 (−43, 57))	191 (150, 233)	12 (−38, 62)	−41 (−93, 11)	48 (−24, 120)	48 (−24, 120)
VO2max (ml/kg/min)	23.8 (22.9, 24.7)	0.69 (−0.42, 1.81)	0.53 (−0.56, 1.62)	24.06 (23.3, 24.9)	0.73 (−0.18, 1.63)	0.35 (−0.56, 1.26)	−0.03 (−1.47, 1.40)	0.18 (−1.24, 1.60)
BMI (kg/m2)	32.7 (31.5, 33.8)	0.37 (0.04, 0.70)*	0.28 (−0.03, 0.60)	31.4 (30.5, 32.4)	−0.08 (−0.41, 0.26)	0.35 (0.03, 0.67)*	0.45 (−0.02, 0.92 )	−0.07 (−0.52, 0.39)
Fatpercentage (%)	39.5 (38.9, 40.0)	−0.16 (−0.71, 0.38)	0.92 (0.40, 1.44)*	38.3 (37.8, 38.9 )	−0.11 (−0.66, 0.45)	0.24 (−0.30, 0.79)	−0.06 (−0.83, 0.72)	0.67 (−0.08, 1.43)
Waistcircumference (cm)	96.2 (94.2, 98.1)	−1.8 (−3.2, -0.37)*	−2.06 (−3.42, -0.70)*	94.2 (92.3, 96.0)	−1.49 (−2.8, -0.17)	−1.29 (−2.6, -0.01)	−0.31 (−2.27, 1.64)	−0.77 (−2.64, 1.11)
Mental well-being	15.8 (14.1, 17.4)	−0.06 (−1.07, 0.95)	0.35 (−0.62, 1.32)	19.5 (18.0, 20.9)	0.24 (−0.68, 1.16)	−0.00 (−0.96, 0.96)	−0.30 (−1.67, 1.07)	0.35(−1.01, 1.72)

Table 2 Changes in the Exercise on Prescription and the Control Group. Δ 6 months: changes at 6 months compared to baseline, Δ 12 months: changes at 12 months compared to baseline. *; significant difference (*P <* 0.05).

#### Physical activity whilst commuting

Within the EoP group, the amount of PA during commuting increased at 6 months and the amount of PA during work decreased at 12 months (Table 2).

#### Physical activity in leisure time

**Figure 2:**
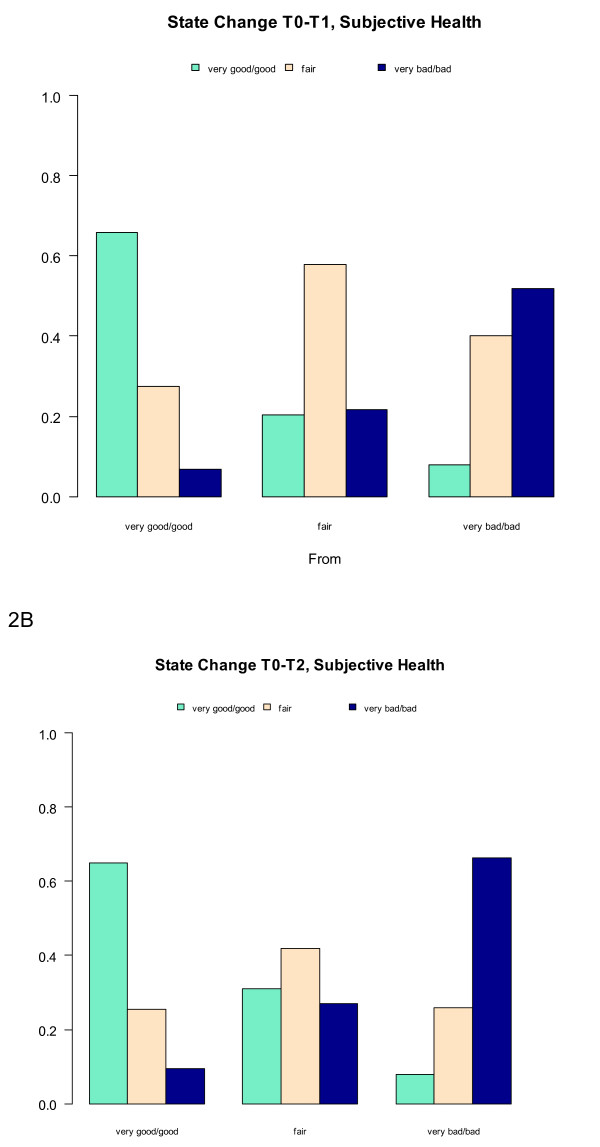
**Changes in subjective health in the Exercise on Prescription group. 2A**: State changes in subjective health from baseline (T0) to 6 months (T1). On the x-axis the rated subjective health at baseline (very/very good, fair, very bad/bad). Per outcome of subjective health at baseline the state of subjective health at 6 months is visualized in the 3 bars. **2B**: State changes in subjective health from baseline (T0) to 12 months (T2). On the x-axis the rated subjective health at baseline (very/very good, fair, very bad/bad). Per outcome of subjective health at baseline the state of subjective health at 6 months is visualized in the 3 bars.

The change in the amount of PA during leisure time differed significantly between the EoP and control groups at 6 and at 12 months. At both time points, the control group became significantly physically less active during leisure time whereas the EoP group stayed at the same PA level (Figure 2, Table 2).

#### Physical activity during household activity

The control group also became physically less active during household activity at 6 and 12 months, whereas the EoP became more active at 12 months (Table 2).

### Physical fitness

EoP had no effect on oxygen uptake. At 6 months BMI had increased significantly within the EoP group (Table 2) and at 12 months fat percentage had increased by 0.9% within the EoP group, whereas waist circumference decreased within the EoP group, -1.8 cm at 6 months and −2.06 at 12 months. However, waist circumference also decreased in the control group, -1.49 cm at 6 months and −1.29 cm at 12 months. In none of these variables were significant differences found between the changes in the EoP and control groups.

### Mental well-being

Mental well-being and subjective health did not change in either of the groups (Table 2, Figure 2a and b).

### Use of care

No significant changes were found within the EoP group or between the EoP group and the control group for the use of care. (Table 3).

**Table 3:** Use of Care

	**N**		**Baseline**		**6 Months**		**12 Months**	
	**EoP**	**C**	**EoP**	**Control**	**EoP**	**Control**	**EoP**	**Control**
Contact general practitioner	91	165	82%	76%	70%	71%	75%	73%
Contact specialist	90	162	43%	40%	52%	36%	49%	38%
Contact physiotherapist	89	164	60%	44%	54%	40%	51%	34%

Table 3.Percentage of care used by the “Exercise on Prescription” Group and the control group at baseline, 6 months and 12 months.

## Discussion

EoP was successful in recruiting its target population and compliance was high. EoP had a small positive effect on PA during leisure time (short-term and long-term) as well as on PA during household activity (long-term), but no effect was found on the total amount of PA. The effect of EoP on total PA, health status and healthcare use was not significant.

Although the target group was reached, the effect of EoP on PA, health status and healthcare use was very small. This is not in accordance with many other ERS programs. However, there is an important difference with other ERS programs in that our target group consisted of physical inactive women from a multi-ethnic population living in deprived neighborhoods. It is known that these women more frequently face complex socioeconomic problems like poverty, unemployment, social isolation and mental illness. Therefore, the limited effects that we found in our study could possibly be explained by these problems, as incorporation of PA in daily life may not have taken priority.

Moreover, compared to other ERS programs, the training frequency of only once a week was low. We are aware of the fact that the frequency of just one single training session a week is probably too low to expect changes in aerobic fitness, weight loss or waist circumference [24], although results of previous studies on the association between intensity of the intervention and its effects are mixed [9]. However, our expectation was, based on social cognitive models [25], that EoP would induce an increase in total PA and that this increase together with the single weekly training session would cause an improvement in health status. Unfortunately, this was not the case. This is probably not only related to the socio-economic problems the target group faces, but also to the fact that 60% of these women were obese. Morgan et al. [8] concluded in their review on evidence for exercise referral schemes, that these programs can improve PA levels at short-term in those who are overweight but not in those who are obese. A reason for that might be that the EoP program, like most other EoP programs, focused just on changing PA levels and not on healthy food patterns.

The EoP dropout rate was only 14%. Compared to other literature this is a very low number [12,26,27], for example, Gidlow et al. [26] reported in their review on attendance of ERS in the UK an adherence level of approximately 20%. EoP was developed to meet the environmental, economic and cultural needs of migrant women living in deprived neighborhoods in the Netherlands, as described by Schmidt et al. [16]. Hence, women were referred by their GP, training sessions were held in their neighborhood in a supportive environment under the supervision of a female coach and financial incentive was available. These factors may be part of the success of the program in reaching its target group and in the adherence to the program. The low dropout rate may, however, also be an explanation for not finding substantial effects of EoP. Perhaps in other studies only the motivated participants who managed to incorporate a healthier lifestyle finished the program, which then led to positive effects. For motivation of the participants seems to be the key factor for the effectiveness this kind of programs [9].

### Limitations

The recruitment of the intervention group was a challenge. Multiple factors which have been reported in the literature, such as the GP’s collaboration and the cooperation of a difficult target population were of influence [28]. In particular in view of the insufficient recruitment of the participants by the GPs, we decided to leave our initial protocol and to make use of the natural patient flow of the EoP program for our intervention group. Due to the different selection processes, we observed differences in the health status of the intervention and the control group. More specifically, the health of the EoP group was worse than that of the control group: they had a higher prevalence of obesity, had a poorer mental well-being, a lower subjective health and a poorer fitness level. One of the explanations for this finding could be the selective referral of women by their GP (confounding by indication), perhaps only the women with obvious health problems were referred. Another explanation could be that women with severe health complaints who were referred to the control group refused to participate in the study. We do not expect these differences between the intervention and control group to have influenced the results, however, as these differences have been controlled for in the statistical analyses.

Another possible limitation might be related to the measurement of the primary and secondary outcomes. This includes the use of self-reported data on physical activity, and the use of a single item measurement of self-perceived health. Participants might have over- or underreported their actual level of physical activity, and the reliability of a single item measurement for self-perceived health might be limited. We do not expect these biases to be substantial, however, given that we found the results to be consistent over a broad range of outcome measures, measured with different types of instruments.

## Conclusions

EoP was successful in including its target population and compliance was high. The effect of EoP on PA, health and mental well-being was limited. It seems that the combination of a socially disadvantaged target group with a high prevalence of obesity and the low intensity of the training program were responsible for not finding positive effects of EoP on total PA, health status and healthcare use. Whether EoP will be effective after the implementation of a dietary component and a more intensive exercise program remains to be investigated. In sum, in this format EoP was not able to increase the PA levels and health status of non-Western migrant women living in deprived areas in the Netherlands.

## Abbreviations

BMI: Body mass index; EoP: Exercise on Prescription; ERS: Exercise referral schemes; IPAQ: International Physical Activity Questionnaire; PA: Physical activity; SQUASH: Short questionnaire to asses health enhancing physical activity.

## Competing interests

The authors declare that they have no competing interests.

## Authors’ contributions

MD, KH and KS developed the concept of the current study. All authors were involved in the coordination of the data collection. MG conducted the analyses. MG and KS interpreted the results. All authors contributed to the writing of the manuscript and all have read and approved the final version.

## Pre-publication history

The pre-publication history for this paper can be accessed here:

http://www.biomedcentral.com/1471-2458/12/758/prepub
